# Fat Replacement by Vegetal Fibres to Improve the Quality of Sausages Elaborated with Non-Castrated Male Pork

**DOI:** 10.3390/ani10101872

**Published:** 2020-10-14

**Authors:** Macarena Egea, Daniel Álvarez, Irene Peñaranda, Nuria Panella-Riera, María Belén Linares, María Dolores Garrido

**Affiliations:** 1 Department of Food Science and Technology, Veterinary Faculty, University of Murcia, 30071 Murcia, Espinardo, Spain; macarena.egea@um.es (M.E.); dalvarez@um.es (D.Á.); irene.penaranda@um.es (I.P.); blinares@um.es (M.B.L.); 2 IRTA-Monells, Product Quality Program, Finca Camps i Armet, 17121 Monells, Girona, Spain; nuria.panella@irta.cat

**Keywords:** fat-reduced, sausage, boar taint, non-castrated male pork, vegetable fibre

## Abstract

**Simple Summary:**

Traditionally, male pigs were castrated without anaesthesia or pain relief before they reached 7 days of age to avoid the production of an undesirable odour and flavour in their meat, termed “boar taint.” In recent years, to improve animal welfare according to European recommendations, farmers have generally abandoned this practice, increasing the supply of non-castrated male pork in the market. Based on this, alternatives are required to improve the quality of meat and meat products derived from these animals, as these products also possess different texture characteristics that make the meat harder and less juicy due to the presence of less fat. Recently, health has become more important to consumers, and reducing the fat content in non-castrated male pig products by replacing it with vegetable fibre has been shown to represent a good strategy for masking and improving texture.

**Abstract:**

Based on the need to find alternatives for the use of meat from non-castrated male pigs that contains high levels of androstenone and skatole, the production of meat products (raw and Frankfurt sausages) with reduced fat content was proposed, as these compounds are lipophilic. For this purpose, three batches of each product (total six) were produced. These included a control batch (1); normal fat content and two fat-reduced batches, where (2) fat was replaced with inulin and β-glucan, or (3) fat was replaced with inulin and β-glucan in addition to a skin grape by-product. These groups used meat from non-castrated male pigs that contained 6.25 µg/g androstenone and 0.4451 µg/g skatole. In general, fat-reduced sausages exhibited less brightness than did the controls. The texture results in Frankfurt were similar to those of the control, while Spanish sausage supplemented with grape skin exhibited reduced hardness. Both strategies resulted in a reduction in boar taint, and this reduction was up to 87.3% in raw sausages with grape by-products. Fat reduction could provide an interesting strategy to allow for the use of tainted meat from non-castrated male pigs.

## 1. Introduction

Currently, animal welfare is an issue that is increasingly becoming a consumer concern. These consumer concerns, in turn, pressure policy-makers to act by imposing stricter regulations on the methods by which animals are reared and treated [1]. This is particularly true in regard to the new European Union recommendations that instruct farmers to abandon certain practices such as tail docking, tooth reduction, and surgical castration [2]. Castration without anaesthesia or pain relief was primarily introduced to prevent boar taint, which results in an unpleasant odour and flavour. Androstenone (a male sex pheromone associated with urine and perspiration odour) and skatole (a metabolite of the amino acid tryptophan associated with naphthalene and faecal odour) are primarily responsible for boar taint-related off-flavours in pork [3]. Abandoning this practice has resulted in the recurrence of this organoleptic disadvantage. In addition, similar to the production of dry-cured ham, producing raw-fermented products from non-castrated males may result in some disadvantages. Due to the lower fat content of male pig meat resulting in a low water-holding capacity, further processing water losses result in drier, harder, saltier and darker final products [4]. Based on this, the issue of promoting meat valorization must be addressed. For this purposs, it is important to develop alternative uses for this meat to allow boar meat to be more widely sold on the national and international markets [5]. Androstenone is non-polar and predominantly fat-soluble, while skatole is both water- and fat-soluble [3]. Based on this, meat products that are typically elaborated with high percentages of fat (20–30%) could present high concentration of boar taint- causing compounds, and then, more undesirable odour and flavour [4].

However, fat reduction of meat products usually implies the occurrence of undesirable technological effects and structural and sensory characteristics such as a reduction in yield, negative changes in texture, and a reduction in consumer acceptance [6]. Therefore, alternatives to improve these deficiencies are required. The growing emphasis on health has led many investigators to explore the feasibility of substituting animal fat with vegetable fibres in various meat products [7]. The use of dietary fibre results in a healthier meat product, and it is also an alternative ingredient that can maintain the technological and sensory properties [8].

These fibre ingredients are non-digestible polysaccharides that can be obtained from vegetables [4], edible mushrooms [9], and microorganisms [10]. One example is inulin, a soluble dietary fibre with different levels of polymerisation. For example, up to sixty monomers of fructose can be bound by β-2-1 glycosidic linkages [8]. This compound is used in numerous food formulations to improve some characteristics of meat products such as water retention capacity and stability of the emulsion [11]. β-Glucan is another non-starch polymer that is primarily composed of the linear polysaccharide (1→3), (1→4)-β-D-glucan and can be obtained from vegetables or mushrooms [12]. The addition of cereal β-glucans to food matrices has been demonstrated as beneficial in modifying the textural properties and improving the stability of emulsions during storage, and these benefits are based mainly on the ability of these compounds to increase the viscosity of aqueous solutions and to form stable gels [9]. Finally, Álvarez et al. [7] demonstrated the use of vegetable by-products such as rice bran or walnuts to improve the textural quality and gelling properties of meat products. Therefore, the use of these vegetable fibres results in an increase in juiciness, can prevent drying during cooking, and can improve the emulsifying capacity, water retention capacity, and the stability of the meat mass to ultimately reduce the hardness and fracturability to enhance the cohesiveness of the product [13].

In the literature revised, it was not shown the use of vegetable fibre to improve valorization of products elaborated with meat from non-castrated male pigs with high androstenone and skatol levels. So, the aim of this study was to investigate the use of inulin, β-glucan, and grape fibre as fat replacers during low-fat meat production (Frankfurt and Spanish style sausages) and to evaluate the effects of these additives on technological parameter changes and their contribution to the reduction of boar-taint perception.

## 2. Materials and Methods

### 2.1. Meat Samples

**Figure 1:**
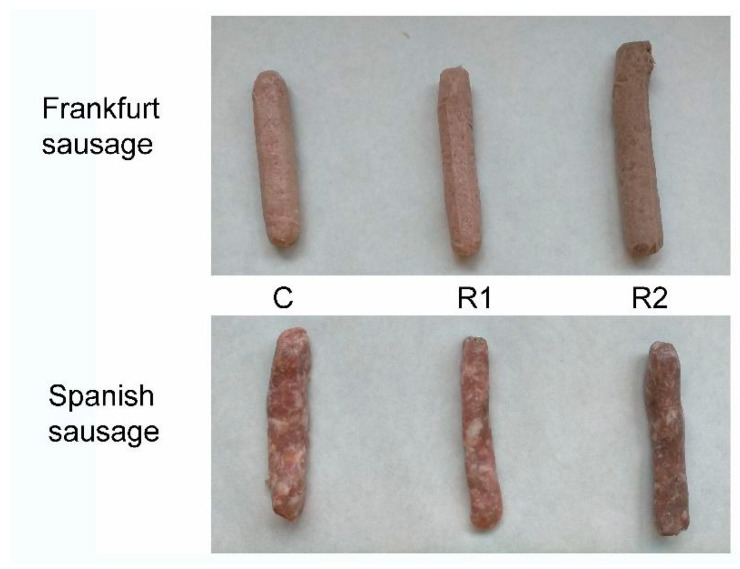
Frankfurt and Spanish sausage and formulations. C: control regular fat content; R1: fat reduced with inulin + β-glucan; R2: fat reduced with inulin + β-glucan + grape skin.

Thirty-one carcasses with higher boar taint were selected using a simple sensory evaluation (human-nose method) from among three hundred animals from a slaughterhouse located in Catalonia (Spain) [14]. An androstenone analysis was performed using the gas chromatography-mass spectrometry (GC-MS) technique and a skatole analysis by high-performance liquid chromatography (HPLC) following Borrisser-Pairó et al. [14]. From among the four animals with the highest level of boar taint compound, one animal was finally selected with the higher level of boar taint. Its meat contained 6.25 µg/g androstenone and 0.451 µg/g skatole. Lean pork ham, (raw fat content 1.25% (*w/w*)) and fat (raw fat content 71.98% *(w/w*)) was used. Two types of sausages were elaborated (Figure 1): Frankfurt sausages (cooked product, needs to be heated before consumption) and Spanish sausages (fresh product, needs to be cooked before consumption). For each type of product, it was proposed three formulations. The control formulations were: 25% pork back fat: 75% lean ham for Frankfurt control (FC) and 30% pork back fat, and 70% lean ham for Spanish sausage control (SC). The remaining two formulations were composed using 12.5% fat in Frankfurt and 20% in Spanish sausages. Inulin (Guinama, Barcelona, Spain), β-glucan (Guanjie Biotech, Shaanxi, China), and red grape skin (Cimusa-Dallant, S.A., Murcia, Spain) were added in the following proportions: FR1: 6% (*w/w*) inulin, 0.5% (*w/w*) β-glucan; FR2: 3% (*w/w*) inulin, 1% (*w/w*) β-glucan, 0.5% (*w/w*) grape pomace, SR1: 3% (*w/w*) inulin, 0.5% (*w/w*) β-glucan, and SR2: 6% (*w/w*) inulin, 1% (*w/w*) β-glucan, 0.5% (*w/w*) grape skin. The detailed composition of each emulsion is provided in Table 1.

### 2.2. Frankfurt Sausage Manufacturing

Excessive connective tissue of raw meat and fat was removed prior to grounding the ham and fat, respectively, through a 25–10 mm plate. After weighing all the ingredients, including lean meat, fat, inulin, β-glucan, grape extract, commercial mix (salt, cornstarch, vegetable fiber, dextrose, smoke aroma, spices and spice extract. Phosphates (E-451), monosodium glutamate ((E-621), sodium ascorbate (E301) and sodium citrate (E-331), sodium nitrates (E-250), carmine (E-120); Catalina Food Solutions S.L., Murcia, Spain), salt (Aliada, Madrid, Spain), and water, the raw mix ingredients were chopped with ice in a high-speed cutter (Robot Coupe R 5 V.V., Borgoña, France). The ingredients were added gradually into the cutter, where the meat batter was formed and the meat emulsion was elaborated. The meat batter was then deposited into a manual stuffer (Garhe S.A, Vizcaya, Spain) and stuffed into 15 mm diameter collagen casings (Edicas, Girona, Spain). Next, the frankfurters were cooked in a scalding kettle (Teycomur, Murcia, Spain) at a ramp temperature of 90 °C for 40–45 min until the core of the sample reached 72 °C (Testo 925, Barcelona, Spain). After thermal treatment, all frankfurter sausages were chilled at 4 °C. The next day, the casings were removed, and the sausages were vacuum-packed and frozen at −18 °C until use. Two batches were produced within one week time following the same process (replicates).

### 2.3. Spanish Sausage Manufacturing

Once the meat and fat were conditioned, all the ingredients necessary for the preparation were weighed. Then, the mixing of ingredients was performed manually until the dough was highly homogeneous and the dough was then stored in the refrigerator (4 °C). The meat batters from three formulations were filled into natural lamb casings possessing a 20 mm diameter (Botía Butcher, Murcia, Spain) using a manual filler (Garhe S.A., Vizcaya, Spain). Finally, sausages from each formulation were weighed for later weight control and then vacuum-packed, and frozen at −18 °C in a freezing chamber. Two batches were produced within one week time following the same process (replicates).

### 2.4. Instrumental Colour

The instrumental colour of the sausages was determined using a CR-400 Chroma Meter (Minolta Ltd., Milton Keynes, UK, 8 mm diameter aperture, d/0 illumination system, D65 illuminant, and a 2° standard observer angle) in the CIELab system. L* (lightness), a* (redness), and b* (yellowness) were measured on the cutting surface from three randomly chosen spots of three slices of frankfurters.

### 2.5. Texture

Texture Profile Analysis (TPA) was performed in triplicate through the use of a texture analyser (Brookfield, Harlow, UK) using the software TexturaPro CT V1. 8 (Build 31) according to the procedures described by Sousa et al. [15] and de Ávila et al. [16]. Measurements were performed at an ambient temperature of approximately 22 °C. The sausages were cut into cylinders that were 2 cm in diameter and 2 cm wide. A double compression cycle test was performed at up to 50% compression of the original portion height with a cylinder probe that was 10 mm in diameter. Force-time deformation curves were obtained with a 25 kg load cell applied at a cross-head speed of 2 mm * s^−1^ and a 5 g trigger point. According to Bourne [17], the measured parameter settings were hardness, adhesiveness, chewiness, gumminess, cohesiveness, elasticity, and resilience. 

### 2.6. Sensory Analyses

Eight panellists from the Department Staff of Food Technology of the University of Murcia that possessed experience in profile assessment of different meat products were selected and trained following the procedure described by Garrido et al. [18], with some modifications to incorporate skatol. Three theoretical-practical sessions of 1.5 h were held for specific training in regard to reduced fat sausages elaborated with meat from castrated males (commercial product), and the other three sessions were performed for training regarding meat products from non-castrated male pigs. A quantitative descriptive analysis (QDA) test was performed using a structured 10 cm non-structured scale. The sensory evaluation of the meat was carried out according to ISO 4121 [19] in a standardised room in the Department of Food Technology (ISO 8589. [20]). The analyses were performed during different morning sessions at 10:30 AM, and a total of six samples were analysed per panellist in each session.

Each panellist tasted a total of three samples per treatment and replicate. After removing the respective end points, the frankfurters were divided into slices (2 cm thick). Two slices were placed in a glass Petri plate (50 × 14 mm) and were heated in a microwave (4 s, 800 W) until the internal temperature reached 72 °C (Hanna Instruments, Woonsocket, RI, USA). The samples were immediately served to the panellists. According to Garrido et al. [4], each panellist opened the Petri plate, sniffed the sample, and then tasted it. Water and unsalted bread were provided to cleanse the palate of residual flavour notes between samples. Sample presentation was balanced to account for order and carryover effects [21].

The evaluated attributes included (Table 2) colour (“colour intensity”, “brightness”, and “homogeneous colour”), intensity of different odours (“frankfurter odour”, “acid odour”, “boar taint”, and “other odours”), taste (“acid”, “salty”, and “bitter”), and flavours (“frankfurter flavour”, “boar taint”, and “other flavours”). “Other odours” and “other flavours” were assessed as different perceptions with respect to the standard frankfurter and were not associated with androstenone or skatol. Panellists were required to define the “other odours” and “other flavours” they perceived. For textural parameters, “hardness”, “cohesiveness”, “chewiness”, and “juiciness” attributes were evaluated. Additionally, the panellists were asked to express their overall rating of the samples.

### 2.7. Statistics

For the analysis of means of colour and texture, a one-way ANOVA analysis was performed, with different formulations (C, R1 and R2) as fixed effect for each type (Frankfurt and Spanish). For the sensorial data, a one-way ANOVA was performed, considering the effects of formulations (C, R1 and R2) as fixed sources of variation and the session, panellists and replicate as a random effect for each type (Frankfurt and Spanish sausages). It was used SPSS version 24.0 software (SPSS Inc., Chicago, IL, USA). Tukey’s test for normality and independent samples was applied. The significance level was set at *p* < 0.05. For sensory analysis, a principal component analysis (PCA) was used to find relationships between sensory attributes and treatment-type of sausages, individual assessor data were included. For this purpose, Minitab 18 (Minitab LLC., PA, USA) was used.

## 3. Results and Discussion

### 3.1. CIELab Colour

Table 3 provides the lightness (L*), redness (a*) and yellowness (b*) values of Spanish and Frankfurt reduced-fat sausages. There were statistical differences for all parameters, with the exception of the angle hue. There was a reduction in lightness in both products and in response to both strategies (*p* < 0.001). This was expected, as the increase in the fat proportion contributed to an increase in L* value [22] that was likely due to a major light reflection. Šojić et al. [22] also observed this phenomenon in cooked sausages made with 5% inulin, where these sausages exhibited significantly lower L* values than did the controls. The authors of that study showed that colour is a very interesting parameter for cooked meat products, as consumers associate this type of meat product with a bright and characteristic pink colour. Similar results were found by Ryu, Shim, & Shin [23] in a study in which grape skins and seed pomace were added to cooked pork sausages. It was observed that the L* value was lower when doses were higher, although they did not reduce the fat content.

Riazi et al. [24] evaluated the colour of cooked beef sausages that were treated with a lower concentration of nitrites from red grape pomace (2%). This treatment resulted in a product with L*, a*, and b* values that were significantly decreased in comparison to those of the control. In agreement with previous studies, they suggested that browning of sugars and the oxidative browning of the tannins that are present in grape pomace could be responsible for the product darkening. Browning reactions are typically accelerated at high temperatures, and this can result in an increase in the extent of the Maillard reaction that occurs in the sausages containing grape pomace powder. A similar trend was observed in regard to a* by Ryu, Shim and Shin [23] when grape skins and seed pomace were added to cooked pork sausages. Sausages supplemented with 0.5% and 1% grape products exhibited reduced CIELab a* values, and these could be related to the anthocyanins present in grape skin. Similar results were observed by Choi et al. [25] when pork fat was reduced and partially substituted for using a mix of grape seed oil (0%, 5%, 10%, and 15%) and 2% rice bran fibre.

Contrasting results were found by Šojić et al. [22], who observed that cooked sausages made with 5% inulin exhibited a significantly higher a* value compared to that of the control. This was expected, as the increase in the fat proportion contributed to a decrease in a* value. Additionally, these results were not unique, as they are also observed in this study.

Other authors also observed a reduction in b* after the addition of vegetable fibres [21,24]. Mainente et al. [26] revealed that flavonoids are the molecules that are primarily involved in the colour of grape pomace, and it must be considered that the colour of anthocyanins could vary from red to blue depending on the pH value. However, phenols can influence the colour of meat based on their antioxidant activity, there they primarily act on myoglobin [27] but can also influence lipid oxidation. The variation of a* value appears to be related to lipid oxidation, while changes in the b* value could be correlated to the oxidation of heme [26].

### 3.2. Texture Profile Analysis

Frankfurt sausages exhibited no differences (Table 4) between the control and fibre-replaced groups (*p* > 0.05). Šojić et al. [22] observed similar results in cooked sausages made with 5% inulin, and Han and Bertram [28] observed similar results using 2% inulin. The authors remarked that the results could be explained by the protein content of both groups of sausages, as these contents were highly similar and appeared to play a major role in the tenderness of the cooked sausages. Ryu et al. [23] also did not observe differences in a study where grape skins and seed pomace (0.5% and 1%) were added to Chinese-style cooked pork sausages, and Huang et al. [29] also did not observe any differences.

Other studies have found that hardness is higher in fibre sausages than it is in controls; however, inulin was combined with other fibres such as oat and wheat fibre [30]. It has been reported that insoluble fibre can increase the cohesiviness of meat products by forming an insoluble three-dimensional network that is capable of modifying the rheological property of the continuous emulsion stage [31]. The same results were observed by Selgas et al. [32] when they studied inulin (in powder form and as gel) that was incorporated into the elaboration of reduced-fat (30% less than normal content) cooked meat sausages. In this research, the authors speculate that the greater hardness is due to the lower amount of fat. Despite this, the addition of inulin caused a significant softening of the sausages when it was added in gel form.

Spanish sausages supplemented with grape pomace (SR2) exhibited reduced hardness, gumminess, and chewiness (*p* < 0.05) compared to these values in the controls. Frankfurts were prepared in a smaller minced meat batter than were Spanish sausages, and this resulted in more homogeneous products and could explain the differences between the products. Elleuch et al. [33] (2011) proposed that the effectiveness of various fibres in ground meat mixtures and emulsions is usually affected by particle size and the type of ions present in the system [28]. The different characteristics of dietary fibres, such as molecular weight and hydrophobicity, cause differences in their physicochemical properties, including water solubility, viscosity enhancement, opacity, surface activity, and binding capacity [34]. However, this characteristic could be interesting, as products elaborated with meat from non-castrated male pigs are hardener than those made from castrated pigs, likely due to the lower fat content (and possibly also lower WHC) of the raw material. Based on this, higher processing losses may occur, and this can result in drier and harder final products [4].

These results are similar to those of Wan Rosli et al. [35] who examined chicken meat frankfurter sausages that had their fat replaced by oyster mushroom powder (rich in β-glucan; up to 6%). They observed that the hardness attribute was significantly lower than that of the control. It is possible that the results from the present study are related to the presence of higher levels of β-glucan and grape pomace fibres. Gumminess and chewiness are secondary parameters that depend on the hardness, and based on this, they behave similarly [32]. Selgas et al., [32] observed that a reduction in fat caused a decrease both in the force and in the work of cutting. This decrease in hardness could be related to the presence of fibre incorporated in the form of an aqueous solution. The author observed that the composition of the fibre sausages contained more water (10%) than did sausages manufactured by other authors, who found an increase in these texture parameters. Differences in composition result in a different protein:fat:water ratio. If the ratio is a determining factor in the consistency of the resulting meat gel, it is possible that the higher fat content in Spanish sausages formulation influenced the texture differences that were observed. Additionally, SR2 possessed half the concentration of inulin. This is consistent with the findings of Selgas et al. [32], who reported that there was a tendency to decrease hardness and shear force as the concentration of inulin increased. Additionally, the literature described an inverse correlation between the shear strength of the samples and the amount of white grape pomace powders that were added [26].

Both products exhibited no differences in adhesiveness and resilience (*p* < 0.05). Other studies observed similar results in fat-reduced Frankfurt sausages [31,34].

Cohesiveness and elasticity were not affected by fibre replacement in Frankfurter sausages, while there was reduction in cohesiveness in the Spanish sausages SR1 and SR2 (*p* < 0.05) and in elasticity in SR1. Similar to results observed for Frankfurter sausages, Selgas et al. [32] found that the springiness and cohesion in cooked meat sausages were very similar across all batches. In accordance with this, Wan Rosli et al. [35] found that the addition of up to 6% concentration of oyster mushroom powder led to results that were similar to these of the control or of Barretto et al. [30] when only 0.58% wheat fibre was added.

The cohesiveness of the control samples was generally higher than that of the fibre groups in regard to Spanish sausages. Similar results were found when meat and fish products were enriched with grape pomace [26]. According to Han and Bertram [28], the majority of previous studies have shown that a reduction of fat in emulsified meat products leads to a firmer texture due to the presence of more tight connections among the meat particles and a more dense structure caused by the reduction in fat. This flavours increased hardness, cohesiveness, and chewiness; however, the doses used in this study did not result in any texture (Frankfurt sausages) or a reduction (Spanish sausages) in texture.

### 3.3. Sensory

**Figure 2:**
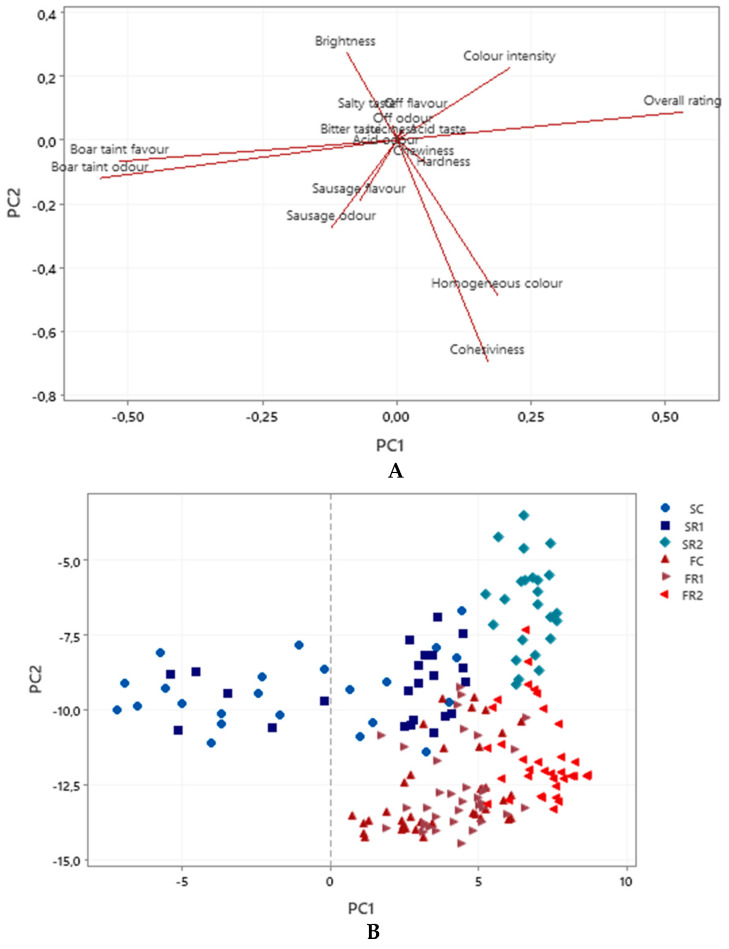
Principal components analyses of sensory test attributes. (**A**): projection of the sensory attributes analyzed. (**B**): projections on the Frankfurt (F) and Spanish (S) sausages with three formulations. C: control regular fat content; R1: fat reduced with inulin+β-glucan; R2: fat reduced with inulin+β-glucan+grape skin.

Figure 2 shows the results delivered by the principal components analysis. Principal component 1 (PC1) was the most relevant accounting for 44.6% of the total variability. The PC1 was positively related with overall rating and colour intensity and negatively related with boar taint odour and boar taint flavour. The results for both R2 formula products placed then on the right side of the chart, SC and SR1 products on the left side, and FC and FR1 products on the right side, but in an intermediate position. SR2 and FR2 took the best position in relation with a low boar taint odour and flavor perception; conversely, Spanish C and R1 score the worst position. Principal component 2 (PC2) explained 24.1% of the total variability. The PC2 was positively related with colour intensity and brightness, and negatively related with cohesiviness, homogeneus colour, sausage odour and flavour. Spanish sausages occupied a less negative area, while Frankfurt sausages (FC, FR1 and FR2) occupied the negative extreme. Frankfurt sausages are related with homogeneous colour and cohesiviness. The R2 sample was closely related to colour intensity, probably due to its grape pomace content.

The sensory evaluation results are presented in Table 5 and Table 6. Reduction of brightness was only perceived by a trained panel in FR2 (*p* < 0.05). SR2 presented higher values than SR1 (*p* < 0.05). Colour changes were only detected on R2 for both products, and these values were higher than those of the control and R1 (*p* < 0.001). As observed from CIElab results, the presence of anthocyanins and tannins from grape pomace could be related to these results.

Morin et al. [36] found that consumers prefer low brightness. Apparently, this was due to consumers associating lighter colour with a higher fat content, or it may simply be that they did not like lighter sausages as much.

Šojić et al. [22] cooked sausages made with 5% of inulin, and they found that the appearance did not differ significantly between groups and also that the colour was significantly darker in the reduced sausages compared to that of the controls. The homogeneity was not affected by these treatments (*p* < 0.05).

Sausage odour and flavour were reduced in both products for R2 treatment, and they were reduced only in odour for FR1 (*p* < 0.05). This parameter measures the intensity of typical sausage odour and flavour in a commercial product with no boar taint. This reduction is related to grape pomace presence in the formulation (R2). In addition, there was an increment in SR1, due to the masking effect of the reduction fat strategy. Similarly, Šojić et al. [22] cooked sausages made with 5% of inulin, and these sausages exhibited a significantly higher score for “odour and taste” attributes than the scores of the controls.

Off odour and flavour were present only in the SR2 group. Mainente et al. [26] remarked that phenols and organic acids typically give rise to sensory properties such as bitterness, astringency, and acidity, and studies have described an ‘intense, typical fermented odour’ of grape skin powder. However, punctuation was low (0.4 and 0.3 in 10 scales), and bitterness was not detected. This could be due to the high protein content in meat-based preparations that could prevent the interaction of phenols with salivary proteins, ultimately reducing astringency [24]. In regard to the principal components of the additives, Selgas et al. [32] concluded that inulin could be incorporated into cooked meat products as powder or preferably as a gel at concentrations of at least 2.5% and 5% without important sensory modifications, and this was in agreement with results presented in our study.

None of the analysed texture parameters exhibited any differences compared to those of the control group, with the exception of FR2, which yielded high hardness and chewiness values. Similar results were observed by Šojić et al. [22] for cooked sausages made with 5% inulin, where they found that the scores for sensory evaluated textural properties for sausages supplemented with vegetal fibre were not significantly different (*p* > 0.05). Inulin exhibits a high capacity to bind water and forms gels that are firm, soft, and stable. Based on this, it is likely that inulin addition contributed to the formation of optimal sensory–evaluated textural characteristics of sausages even when the fat content was reduced, and this was also observed in our assay. In contrast, Selgas et al. [32] found that the presence of inulin in powder caused an increase in hardness.

Boar taint odour and flavour were reduced in FR2 (66.7% and 70.8%, respectively), SR1 (35.7% and 49.1%, respectively), and SR2 (85.7% and 87.3%, respectively; *p* < 0.05). Frankfurt sausages exhibited lower boar taint scores (3.0 and 2.4) than did Spanish sausages (5.6 and 5.5). This is likely due to the Spanish sausages possessing a higher fat content. Additionally, Frankfurt is minced and heated for a longer period of time during the cooking process. Processing and commercial additive mixes can provide a “smoke” aroma. Previous studies have also demonstrated that cooking could reduce boar taint perception [37]. The reduction of boar taint is expected due to reduced formulations had 52% and 34% less of fat (Frankfurt and Spanish sausages respectively), and androstenone and skatole are lipophilic components [3]. Previous studies had demonstrated that using 10% concentrations of tainted meat in products results in high levels of consumer acceptance [38]. In this sense, Mörlein et al. [39] found that up to 33% of meat and fat from carcasses possessing skatole concentrations of up to 0.3 μg/g and androstenone concentrations of up to 3.8 μg/g in melted back fat may be used for the production of Frankfurter-type sausages. This is in accordance with the results of Hemeryck et al. [40], who confirmed that mixing patties with Gilt raw materials processing these into Frankfurter sausages or into restructured ham can potentially reduce rejection by consumers.

The masking sensory capacity was higher for FR2 and SR2. Although some organoleptic characteristics of this strategy are slightly different from those of control products, other studies have observed that the global acceptability (including colour, flavour, tenderness, and odour) of raw and cooked chicken hamburgers was increased by the addition of red grape pomace powders at up to 2% concentration [41]. In support of this, Riazi et al. [24] also indicated that pomace from red grapes at concentrations of 1% and 2% *w/w* improved the sensory properties and particularly the taste of the meat. Therefore, both strategies, and particularly that of R2, appear to provide good options for supplementing meat products from non-castrated male pigs.

## 4. Conclusions

Frankfurt sausages initially exhibited a lower perception of boar taint than did Spanish sausages. The R2 strategy (3% inulin, 1% β-glucan, and 0.5% grape skin) resulted in better masking properties in both products, although with a sausage flavour reduction. It can be concluded that the reduction of fat in Spanish and Frankfurt sausages elaborated with meat from non-castrated male pigs with the replacement of vegetal fibres (inulin, β-glucan, and grape skin) could offer a good strategy to mask boar taint and to provide a texture similar to that of commercial sausages.

## Figures and Tables

**Table 1:** Frankfurt and Spanish sausage formulations (*w:w*, %).

Type/Formulation	Lean	Fat	Inulin	β-glucan	Grape Skin	Salt	Mix	Water
FC	60	33.69	-	-	-	1	4	1.31
FR1	60	16.32	6	0.5	-	1	4	12.17
FR2	60	16.32	3	1	0.5	1	4	14.17
SC	50	40.81	-	-	-	1	4	4.19
SR1	50	26.92	6	0.5	-	1	4	11.58
SR2	50	26.92	3	1	0.5	1	4	13.58

F: Frankfurt sausage. S: Spanish sausage; C: control with regular fat content; R1: fat reduced with inulin+β-glucan; R2: fat reduced with inulin + β-glucan + grape skin.

**Table 2:** Definition of attributes used in sensory analysis.

Attribute	Definition	Scale
Colour intensity	Intensity of sausage colour	(0 pink–5 commercial–10 violet).
Brightness	Reflection of light on the surface of the product.	(0 light–10 dark)
Homogeneous colour	Regarding the distribution of the product colour	(0 not homogeneus–10 totally homogenous).
Sausage odour	Intensity of the perception of the characteristic odour of the evaluated product elaborated with meat from castrated pigs.	(0 totally different–10 totally equal).
Acid odour	Describes the basic odour produced by dilute aqueous solutions of citric acid.	(0 not acid–10 acid)
Off odour	Any smell that cannot be included in sausage odour or sexual odour.	(0 not present–10 strong presence)
Boar taint odour	Intensity of the characteristic odour produced by androstenone and skatole.	(0 not present–10 sample with 7 µg/g androstenone and 1.5 skatol µg/g in vaseline oil)
Acid taste	Describes the basic taste produced by dilute aqueous solutions of citric acid.	(0 not acid–10 acid)
Bitter taste	Describes the basic taste produced by dilute aqueous solutions of caffeine.	(0 not bitter–10 bitter)
Sausage flavour	Intensity of the perception of the characteristic flavour of the evaluated product elaborated with meat from castrated pigs	(0 totally different–10 totally equal).
Off flavour	Any flavour that cannot be included in sausage or sexual flavour.	(0 not present–10 strong presence)
Boar taint flavour	Intensity of the characteristic flavour produced by androstenone and skatole.	(0 not present–10 sample with 7 µg/g androstenone and 1.5 skatol µg/g in vaseline oil)
Hardness:	Force necessary to deform the product between the molars at the first bite	(0 tender–5 commercial sample-10 hard).
Cohesiviness	Mechanical textural attribute relating to the degree to which a food can be deformed before it breaks. Compress the sample with molars and evaluate the amount of deformation before rupture.	(0 not cohesive–10 cohesive commercial sample)
Chewiness	Number of chews required to swallow a product.	(0 less chews–5 number of chews needed for commercial product–10 more chews)
Juiciness	Parameter that measures the amount of water released by the product in the first bites.)	(0 not juicy–5 juiciness of commercial product–10 high juicy)
Overall rating	Level of masking of the strategies used in the elaborated products.	(0 no masking–10 total masking).

**Table 3:** Colour CIELAB means ± sd of Frankfurt and Spanish sausages.

**Frankfurt Sausages Treat**				
**Instrumental Colour**	**FC**	**FR1**	**FR2**	***p*-value**
L*	51.6 ± 3.28 ^a^	44.9 ± 0.84 ^b^	42.82 ± 0.69 ^b^	0.000
a*	11.0 ± 0.93 ^a^	8.7 ± 1.48 ^b^	7.8 ± 0.45 ^b^	0.000
b*	6.6 ± 0.91 ^a^	5.7 ± 0.66 ^b^	5.3 ± 0.39 ^b^	0.003
**Spanish Sausages**				
**Instrumental Colour**	**SC**	**SR1**	**SR2**	***p*-value**
L*	54.3 ± 2.47 ^a^	46.6 ± 1.36 ^b^	41.5 ± 0.71 ^c^	0.000
a*	10.4 ± 1.08 ^a^	10.4 ± 1.83 ^a^	7.5 ± 0.24 ^b^	0.000
b*	6.9 ± 0.86 ^a^	6.4 ± 0.87 ^a^	5.0 ± 0.16 ^b^	0.000

F: Frankfurt sausage. S: Spanish sausage; C: control regular fat content; R1: fat reduced with inulin + β-glucan; R2: fat reduced with inulin + β-glucan + grape skin. L*: Lightening, a*: red-green; b*: yellow-blue. ^a^, ^b^, ^c^: Tukey’s test *p* < 0.05.

**Table 4:** Instrumental texture means ± SD of Frankfurt and Spanish sausages.

**Frankfurt Sausage**				
**Parameters**	**FC**	**FR1**	**FR2**	***p*-value**
Hardness	2788.3 ± 303.5	2659.5 ± 199.6	2706.1 ± 242.7	0.582
Gumminess	1474.2 ± 283.01	1639.8 ± 107.19	1546.9 ± 197.65	0.303
Chewiness	146.3 ± 16.17	148.8 ± 6.59	132.9 ± 13.88	0.055
Adhesiveness	0.52 ± 0.317	0.39 ± 0.228	0.34 ± 0.126	0.303
Resilience	0.31 ± 0.018	0.32 ± 0.019	0.31 ± 0.045	0.305
Extensibility	0.69 ± 0.076	0.59 ± 0.062	0.61 ± 0.087	0.052
Cohesiveness	0.63 ± 0.061	0.63 ± 0.048	0.58 ± 0.041	0.053
Elasticity	8.1 ± 1.11	8.5 ± 0.17	8.6 ± 0.15	0.331
**Spanish Sausages**				
**Parameters**	**SC**	**SR1**	**SR2**	***p*-value**
Hardness	2129.3 ± 1322.89 ^a^	2157.2 ± 205.58 ^a^	1837.5 ± 249.49 ^b^	0.008
Gumminess	1213.0 ± 82.9 ^a^	1100.4 ± 127.2 ^a,b^	973.1 ± 166.1 ^b^	0.006
Chewiness	105.1 ± 9.63 ^a^	91.8 ± 10.10 ^a,b^	80.51 ± 14.56 ^b^	0.002
Adhesiveness	0.42 ± 0.45	0.70 ± 0.074	0.10 ± 0.08	0.239
Resilience	0.30 ± 0.033	0.27 ± 0.035	0.23 ± 0.044	0.056
Extensibility	0.98 ± 0.103 ^a^	0.97 ± 0.121 ^a^	1.47 ± 0.298 ^b^	0.000
Cohesiveness	0.57 ± 0.044 ^a^	0.51 ± 0.033 ^b^	0.48 ± 0.042 ^b^	0.001
Elasticity	8.6 ± 0.21 ^a^	8.24 ± 0.30 ^b^	8.52 ± 0.12 ^a,b^	0.013

F: Frankfurt sausage. S: Spanish sausage; C: control regular fat content; R1: fat reduced with inulin + β-glucan; R2: fat reduced with inulin + β-glucan + grape skin. ^a^, ^b^: Tukey’s test *p* < 0.05.

**Table 5:** Sensory analyses LSM +/- SE of Frankfurt sausages.

Attributes	FC	FR1	FR2	SE	*p*-Value
Colour	5.2 ^a^	5.1 ^a^	7.9 ^b^	0.109	0.000
Brightness	4.6 ^a^	4.6 ^a^	3.8 ^b^	0.117	0.000
Homogeneity	9.7	9.8	9.8	0.081	0.681
Sausage odour	8.2 ^a^	7.7 ^b^	6.8 ^c^	0.130	0.000
Acid odour	0.0	0.0	0.0	0.005	0.546
Off odour	0.0	0.0	0.0	0.005	0.720
Boar taint odour	3.0 ^a^	2.4 ^a^	1.0 ^b^	0.175	0.000
Acid	0.0	0.0	0.0	0.005	0.931
Salty	5.3	5.1	5.4	0.098	0.269
Bitter	0.0	0.0	0.0	0.022	0.812
Sausage flavour	8.1 ^a^	7.9 ^a^	6.9 ^b^	0.125	0.000
Off flavour	0.0	0.0	0.0	0.019	0.642
Boar taint flavour	2.4 ^a^	1.8 ^b^	0.7 ^c^	0.107	0.000
Hardness	5.4 ^a^	5.5 ^a^	6.1 ^b^	0.107	0.000
Cohesiveness	9.2	9.2	9.1	0.098	0.962
Chewiness	5.2 ^a^	5.4 ^a^	5.9 ^b^	0.103	0.000
Juiciness	5.3	5.3	5.1	0.101	0.243
Overall rating	6.2 ^a^	6.7 ^a^	7.7 ^b^	0.192	0.000

F: Frankfurt Sausage. C: control regular fat content; R1: fat reduced with inulin + β-glucan; R2: fat reduced with inulin + β-glucan + grape skin. The *p*-value indicated by *, **, and *** for *p* < 0.01 and *p* < 0.001, respectively; NS *p* > 0.05. ^a^, ^b^, ^c^: Tukey’s test *p* < 0.05.

**Table 6:** Sensory analyses LSM +/- SE of Spanish sausages.

Attributes	SC	SR1	SR2	SE	*p*-Value
Colour	5.4 ^a^	5.0 ^a^	8.4 ^b^	0.114	0.000
Brightness	5.7 ^a,b^	5.6 ^a^	6.1 ^b^	0.119	0.005
Homogeneity	6,7	6.7	7.1	0.116	0.119
Sausage odour	7.6 ^a^	8.1 ^a^	5.27 ^b^	0.152	0.000
Acid odour	0.0	0.0	0.0	0.004	0.556
Off odour	0.0 ^a^	0.0 ^a^	0.5 ^b^	0.044	0.000
Boar taint odour	5.6 ^a^	3.6 ^b^	0.8 ^c^	0.327	0.000
Acid	0.0	0.0	0.0	0.003	0.556
Salty	5.5	5.6	5.8	0.109	0.301
Bitter	0.0	0.0	0.0	0.003	0.556
Sausage flavour	7.4 ^a^	8.1 ^c^	5.6 ^b^	0.139	0.000
Off flavour	0.0 ^a^	0.0 ^a^	0.4 ^b^	0.058	0.000
Boar taint flavour	5.5 ^a^	2.8 ^b^	0.7 ^c^	0.332	0.000
Hardness	5.4 ^a^	5.6 ^a^	5.0 ^b^	0.103	0.002
Cohesiveness	6.2	6.4	5.9	0.102	0.081
Chewiness	5.3	5.3	4.9	0.113	0.059
Juiciness	5.4	5.3	5.2	0.136	0.501
Total point	2.9 ^a^	5.9 ^b^	8.6 ^c^	0.331	0.000

S: Spanish sausage; C: control regular fat content; R1: fat reduced with inulin+β-glucan; R2: fat reduced with inulin + β-glucan + grape skin. SE: Standard error NS: *p* > 0.05. ^a^, ^b^, ^c^: Tukey’s test *p* < 0.05.

## References

[1] Gebska M., Golebiewska B., Hubbard C. (2019). Consumer awareness of the selection of animal products from farms that maintain animal welfare. Econ. Agro Aliment. Food Econ..

[2] (2011). European Declaration on Alternatives to Surgical Castration. http://ec.europa.eu/food/animal/welfare/farm/initiatives_en.htm.

[3] Martínez B., Rubio B., Viera C., Linares M.B., Egea M., Panella-Riera N., Garrido M.D. (2016). Evaluation of different strategies to mask boar taint in cooked sausage. Meat Sci..

[4] Garrido M.D., Egea M., Linares M.B., Borrisser-Pairó F., Rubio B., Viera C., Martínez B. (2017). Sensory characteristics of meat and meat products from entire male pigs. Meat Sci..

[5] Wauters J., Vercruysse V., Aluwé M., Verplanken K., Vanhaecke L. (2016). Boar taint compound levels in back fat versus meat products: Do they correlate?. Food Chem..

[6] Bis-Souza C.V., Ozaki M.M., Vidal V.A.S., Pollonio M.A.R., Penna A.L.B., Barretto A.C.S. (2019). Can dietary fiber improve the technological characteristics and sensory acceptance of low-fat Italian type salami?. J. Food Sci. Technol..

[7] Álvarez D., Xiong Y.L., Castillo M., Payne F.A., Garrido M.D. (2012). Textural and viscoelastic properties of pork frankfurters containing canola–olive oils, rice bran, and walnut. Meat Sci..

[8] Álvarez D., Barbut S. (2013). Effect of inulin, β-glucan and their mixtures on emulsion stability, color and textural parameters of cooked meat batters. Meat Sci..

[9] Apostu P.M., Mihociu T.E., Nicolau A.I. (2017). Technological and sensorial role of yeast β-glucan in meat batter reformulations. J. Food Sci. Technol..

[10] Lara-Fiallos M., Lara-Gordillo P., Julián-Ricardo M.C., Pérez-Martínez A., Benítes-Cortés I. (2017). Avances en la producción de inulina. Tecnol. Quím..

[11] Öztürk B., Serdaroğlu M. (2016). A rising star prebiotic dietary fiber: Inulin and recent applications in meat products. Food Health.

[12] Amini Sarteshnizi R., Hosseini H., Khosroshahi N.K., Shahraz F., Mousavi Khaneghah A., Kamranl M., Komeili1 R., Chiavaro E. (2017). Effect of resistant starch and β-glucan combination on oxidative stability, frying performance, microbial count and shelf life of prebiotic sausage during refrigerated storage. Food Technol. Biotechnol..

[13] Piñero M.P., Parra K., Huerta-Leidenz N., De Moreno L.A., Ferrer M., Araujo S., Barboza Y. (2008). Effect of oat’s soluble fibre (β-glucan) as a fat replacer on physical, chemical, microbiological and sensory properties of low-fat beef patties. Meat Sci..

[14] Borrisser-Pairó F., Panella-Riera N., Zammerini D., Olivares A., Garrido M.D., Martínez B., Gil M., García-Regueiro J.A., Oliver M.A. (2016). Prevalence of boar taint in commercial pigs from Spanish farms. Meat Sci..

[15] Sousa S.C., Fragoso S.P., Penna C.R., Arcanjo N.M., Silva F.A., Ferreira V.C., Barreto M.D.S., Araújo Í.B. (2017). Quality parameters of frankfurter-type sausages with partial replacement of fat by hydrolyzed collagen. LWT.

[16] de Ávila M.D.R., Cambero M.I., Ordóñez J.A., de la Hoz L., Herrero A.M. (2014). Rheological behaviour of commercial cooked meat products evaluated by tensile test and texture profile analysis (TPA). Meat Sci..

[17] Bourne M.C. (1968). Texture profile of ripening pears. J. Food Sci..

[18] Garrido M.D., Egea M., Linares M.B., Martínez B., Viera C., Rubio B., Borrisser-Pairó F. (2016). A procedure for sensory detection of androstenone in meat and meat products from entire male pigs: Development of a panel training. Meat Sci..

[19] ISO (2007). ISO 4121: 2003. Meat and Meat Products. Evaluation of Food Products by Methods Using Scales. Sensory Analysis.

[20] ISO (2007). ISO 8589: 2007: Sensory Analysis—General Guidance for the Design of Test Rooms.

[21] MacFie H.J., Bratchell N., Greenhoff K., Vallis L.V. (1989). Designs to balance the effect of order of presentation and first-order carry-over effects in hall tests. J. Sens. Stud..

[22] Šojić B.V., Petrović L.S., Pešović B.M., Tomović V.M., Jokanović M.R., Džinić N.R., Salitrežić P.P. (2011). The influence of inulin addition on the physico-chemical and sensory characteristics of reduced-fat cooked sausages. Acta Period. Technol..

[23] Ryu K.S., Shim K.S., Shin D. (2014). Effect of grape pomace powder addition on TBARS and color of cooked pork sausages during storage. Korean J. Food Sci. Anim. Resour..

[24] Riazi F., Zeynali F., Hoseini E., Behmadi H., Savadkoohi S. (2016). Oxidation phenomena and color properties of grape pomace on nitrite-reduced meat emulsion systems. Meat Sci..

[25] Choi Y.S., Choi J.H., Han D.J., Kim H.Y., Lee M.A., Kim H.W., Ju-Woon Lee J.W., Hai-Jung Chung H.J., Kim C.J. (2010). Optimization of replacing pork back fat with grape seed oil and rice bran fiber for reduced-fat meat emulsion systems. Meat Sci..

[26] Mainente F., Menin A., Alberton A., Zoccatelli G., Rizzi C. (2018). Evaluation of the sensory and physical properties of meat and fish derivatives containing grape pomace powders. Int. J. Food Sci. Technol..

[27] Jongberg S., Skov S.H., Tørngren M.A., Skibsted L.H., Lund M.N. (2011). Effect of white grape extract and modified atmosphere packaging on lipid and protein oxidation in chill stored beef patties. Food Chem..

[28] Han M., Bertram H.C. (2017). Designing healthier comminuted meat products: Effect of dietary fibers on water distribution and texture of a fat-reduced meat model system. Meat Sci..

[29] Huang S.C., Tsai Y.F., Chen C.M. (2011). Effects of wheat fiber, oat fiber, and inulin on sensory and physico-chemical properties of Chinese-style sausages. Asian-Australas. J. Anim. Sci..

[30] Barretto A.C.D.S., Pacheco M.T.B., Pollonio M.A.R. (2015). Effect of the addition of wheat fiber and partial pork back fat on the chemical composition, texture and sensory property of low-fat bologna sausage containing inulin and oat fiber. Food Sci. Technol..

[31] Cofrades S., López-López I., Solas M.T., Bravo L., Jiménez-Colmenero F. (2009). Influence of different types and proportions of added edible seaweeds on characteristics of low-salt gel/emulsion meat systems. Meat Sci..

[32] Selgas M.D., Cáceres E., García M.L. (2005). Long-chain soluble dietary fibre as functional ingredient in cooked meat sausages. Food Sci. Technol. Int..

[33] Elleuch M., Bedigian D., Roiseux O., Besbes S., Blecker C., Attia H. (2011). Dietary fibre and fibre-rich by-products of food processing: Characterisation, technological functionality and commercial applications: A review. Food Chem..

[34] Cui S.W. (2005). Food Carbohydrates: Chemistry, Physical Properties, and Applications.

[35] Wan Rosli W.I., Maihiza N., Raushan M. (2015). The ability of oyster mushroom in improving nutritional composition, β-glucan and textural properties of chicken frankfurter. Int. Food Res. J..

[36] Morin L.A., Temelli F., McMullen L. (2002). Physical and sensory characteristics of reduced-fat breakfast sausages formulated with barley β-glucan. J. Food Sci..

[37] Peñaranda I., Garrido M.D., Egea M., Díaz P., Álvarez D., Oliver M.A., Linares M.B. (2017). Sensory perception of meat from entire male pigs processed by different heating methods. Meat Sci..

[38] Verplanken K., Wauters J., Vercruysse V., Aluwé M., Vanhaecke L. (2017). Sensory evaluation of boar-taint-containing minced meat, dry-cured ham and dry fermented sausage by a trained expert panel and consumers. Food Chem..

[39] Mörlein J., Meier-Dinkel L., Gertheiss J., Schnäckel W., Mörlein D. (2019). Sustainable use of tainted boar meat: Blending is a strategy for processed products. Meat Sci..

[40] Hemeryck L.Y., Wauters J., Dewulf L., Decloedt A.I., Aluwé M., De Smet S., Fraeye I., Vanhaecke L. (2020). Valorisation of tainted boar meat in patties, frankfurter sausages and cooked ham by means of targeted dilution, cooking and smoking. Food Chem..

[41] Sayago-Ayerdi S.G., Brenes A., Goñi I. (2009). Effect of grape antioxidant dietary fiber on the lipid oxidation of raw and cooked chicken hamburgers. LWT.

